# Towards Age-Related Anti-Inflammatory Therapy: Klotho Suppresses Activation of ER and Golgi Stress Response in Senescent Monocytes

**DOI:** 10.3390/cells9020261

**Published:** 2020-01-21

**Authors:** Jennifer Mytych, Przemysław Sołek, Agnieszka Będzińska, Kinga Rusinek, Aleksandra Warzybok, Anna Tabęcka-Łonczyńska, Marek Koziorowski

**Affiliations:** Department of Animal Physiology and Reproduction, Institute of Biology and Biotechnology, Collegium Scientarium Naturalium, University of Rzeszow Werynia 2, 36-100 Kolbuszowa, Poland; pp.solek@gmail.com (P.S.); agnieszka.bedzinska96@gmail.com (A.B.); rusineczek96@gmail.com (K.R.); olawarzybok@vp.pl (A.W.); annaurz@wp.pl (A.T.-Ł.); mkozioro@ur.edu.pl (M.K.)

**Keywords:** klotho, monocytes, immunosenescence, SASP, ER stress response, Golgi apparatus/complex stress response

## Abstract

Immunosenescence in monocytes has been shown to be associated with several biochemical and functional changes, including development of senescence-associated secretory phenotype (SASP), which may be inhibited by klotho protein. To date, it was believed that SASP activation is associated with accumulating DNA damage. However, some literature data suggest that endoplasmic reticulum and Golgi stress pathways may be involved in SASP development. Thus, the aim of this study was to investigate the role of klotho protein in the regulation of immunosenescence-associated Golgi apparatus and ER stress response induced by bacterial antigens in monocytes. We provide evidence that initiation of immunosenescent-like phenotype in monocytes is accompanied by activation of CREB34L and TFE3 Golgi stress response and ATF6 and IRE1 endoplasmic reticulum stress response, while klotho overexpression prevents these changes. Further, these changes are followed by upregulated secretion of proinflammatory cytokines, which final modification takes place exclusively in the Golgi apparatus. In conclusion, we provide for the first time evidence of klotho involvement in the crosstalk on the line ER-Golgi, which may, in turn, affect activation of SASP. This data may be useful for a novel potential target for therapy in age-related and chronic inflammatory conditions.

## 1. Introduction

Aging continuously remodels the immune system, a process known as immunosenescence. Under conditions of chronic psychological stress, bacterial infections or exposure to certain persistent viral infections a so-called premature immunosenescence in younger individuals may occur [1,2]. In our previous papers, we have reported the possibility of initiation of premature immunosenescence in monocytes due to the treatment with bacterial antigens, which leads to several biochemical and functional changes [3,4]. Among these changes, the development of senescence-associated secretory phenotype (SASP) is observed. SASP is characterized by increased release of many factors into the cellular environment, including cytokines and pro-inflammatory chemokines, growth factors, or proteases and their modulators [3,4]. Considering the fact that prematurely senescent cells prolong their lifespan, do not undergo apoptosis, and retain their metabolic and secretory activity, SASP initiation is unfavorable and may lead to many abnormalities, including the development of chronic inflammation as a consequence of an imbalance between the amount of produced pro-inflammatory and anti-inflammatory factors. At present, it is believed that the main contribution of SASP formation is accumulating DNA damage, which results in the mobilization of ATM and CHK2 kinases [5] and NF-κB activation, necessary for increased secretion of pro-inflammatory factors. Interestingly, the literature suggests that an important role in SASP development in prematurely immunosenescent monocytes may also play endoplasmic reticulum (ER) and Golgi apparatus, organelles taking part in the processing, sorting, and exporting of biosynthetic charges. In addition, ER as well as Golgi apparatus act as sensors for cellular stress and participate in the modulation of intracellular signaling pathways responsible for maintaining homeostasis in response to emerging stress.

On the other hand, klotho protein plays an important role in the proper systemic development and functioning of the immune system. Klotho is a type-I membrane protein related to β-glucuronidases that is encoded by the KL gene in humans. Klotho function has been primarily linked with its role as co-receptor of fibroblast growth factor 23 (FGF23) to regulate phosphate homeostasis [6]. Recent studies indicated that klotho might have critical roles in oxidative stress, glucose metabolism, cell proliferation, apoptosis, autophagy, ER stress, and Golgi apparatus stress [7,8,9]. Klotho was also reported to serve as an anti-inflammatory modulator since its depletion contributes to increased inflammation. In a previous paper, we showed how klotho plays a protective role in monocytes against the initiation of bacterial antigens-induced premature immunosenescence and the development of SASP, probably due to the inhibition of DNA damage [3]. Nevertheless, the role of klotho protein in other possible mechanisms of SASP development including Golgi and ER related stress pathways remains undiscovered.

Therefore, the aim of this study is to investigate the role of klotho protein in the regulation of immunosenescence-associated Golgi apparatus and ER stress response induced by bacterial antigens in monocytes.

## 2. Materials and Methods

### 2.1. Materials

All reagents, unless otherwise stated, were purchased from Sigma-Aldrich (Poznan, Poland) and had analytical grade purity.

### 2.2. Cell Culture, Klotho Overexpression, and LPS/Monensin Sodium Salt Treatment

This is a follow-up project utilizing a model of klotho overexpression obtained in our previous study [3], however with a new batch of cells obtained from EACC (Sigma, Poznan, Poland). Human monocytic leukemia cells i.e. THP-1 cells were cultured in RPMI-1640 (Roswell Park Memorial Institute 1640 medium) supplemented with 2 mM L-glutamine, 10% heat-inactivated fetal bovine serum, and standard antibiotic-antimycotic mix solution (100 U/mL penicillin, 0.1 mg/mL streptomycin, 0.25 µg/mL amphotericin B) in a humidified atmosphere in the presence of 5% CO_2_ and at 37 °C. Typically, cells were passaged every 48 h by direct dilution in complete culture medium to the starting density of 1 × 10^5^ cells/mL. Klotho overexpression was obtained by plasmid transfection. Plasmids were gifts from Hal Dietz (Addgene depository plasmid #17712 – membrane form of klotho, #17713 – secrete form of klotho), while control plasmid was obtained from Evrogen (Moscow, Russia) (#FP401). Cells were seeded into 24-well plates at the density of 0.5 × 10^5^ cells/mL in 0.5 mL of complete growth medium. The next day, 0.5 µg of DNA was diluted in RPMI without serum and 0.5 µL Plus Reagent (Invitrogen, Carslbad, CA, USA) was added. After 15 min incubation, 2 µL of Lipofectamine LTX (Invitrogen, Carslbad, CA, USA) was added and incubated for another 25 min at room temperature. Next, 100 µL of the DNA-Lipofectamine complexes was added directly to well-containing cells. On the next day, medium was changed with RPMI supplemented with 1 mg/mL neomycin and the culture was continued for the approximately two months to obtain stable clones (medium with neomycin was changed every three days). Klotho induction efficiency was controlled as previously described. Briefly, induction of membrane form of klotho was controlled with Western Blot. Additionally, klotho secretion to cell culture medium was evaluated: medium was collected and concentrated in a Microsep Advance Centrifugal Device 10 K MWCO (15,000× *g*, 15 min, 4 °C) (New York, NY, USA) and proceeded with Western Blot (loading control was done with simple Coomassie brilliant blue staining using standard protocol) [2]. Cells transfected with plasmids were designated as follows: vector (control plasmid), sKL-pDNA (secrete form of klotho plasmid-plasmid DNA), and mKL-pDNA (membrane form of klotho plasmid-plasmid DNA). Vector, sKL-pDNA and mKL-pDNA THP-1 cells were seeded at density of 1 ×10^5^ cells/mL and treated with 1 µg/mL LPS (lipopolysaccharide) from *Escherichia coli* serotype 0055:B5 for 168 h (every 48 h cells were passaged to starting density of 1 × 10^5^ cells/mL and fresh culture medium with LPS was added) or 0.5 µM monensin sodium salt for 72 h (as positive control for Golgi stress response activation).

### 2.3. Protein Purification and Western Blot

Protein lysates were prepared according to a prior study [3]. Briefly, cells were lysed in a RIPA buffer (radioimmunoprecipitation assay buffer) (50 mM Tris hydrochloride pH 7.5, 1% NP-40, 0.5% sodium deoxycholate, 0.1% SDS, 150 mM NaCl, 1 mM PMSF (phenylmethylsulfonyl fluoride), and 1 mM EDTA (ethylenediaminetetraacetic acid)). After 30 min homogenization in 4 °C, total homogenates were cleared by centrifugation (15,000× *g*, 15 min, 4 °C) and supernatants were moved to fresh tubes. Total protein content was measured using a BCA assay (bicinchoninic acid assay) according to the manufacturer’s protocol (Thermo Fisher, Waltham, MA, USA). Then, 30 µg of lysates were resolved by using 10% SDS-PAGE (sodium dodecyl sulfate–polyacrylamide gel)electrophoresis, which was then transferred onto PVDF (polyvinylidene fluoride) membrane. Membranes were blocked in 1% BSA (bovine serum albumin) in TBST (tris-buffered saline with Tween 20; 20 mM tris hydrochloride pH 7.5, 137 mM sodium chloride, 0.1% Tween 20) at room temperature for 1 h and incubated overnight at 4 °C with the specific primary antibody. The primary antibodies used were: anti-ACTB (anti-β-actin) (1:10,000; #PA1-16889, RRID: AB_568434), anti-klotho (#PA5-21078; RRID: AB_11153007), and anti-O-GlcNAc (anti-O-GlcNAcylation) (#MA1-072; RRID: AB_326364) (Thermo Fisher, Waltham, MA, USA). Other antibodies used were:

- Golgi stress pathway: anti-TFE3 (anti-transcription factor binding to IGHM enhancer 3) (1:500; #PA5-54909, RRID:AB_2648409), anti-HSP47 (anti-heat shock protein 47) (1:1000; #PA5-14254, RRID:AB_2285672), anti-CREB34L (anti-cyclic AMP response element binding 34L) (1:750; #PA5-18028, RRID:AB_10982190), anti-ARF4 (anti-ADP-ribosylation factor 4) (1:1000; #PA5-37841, RRID:AB_2554449), anti-SIAT4A (anti-sialyltransferase 4A) (1:2000; #PA5-21721, RRID:AB_11154540), anti-giantin (1:1000; #PA5-42884, RRID:AB_2607822), anti-WIPI1 (anti-WD repeat domain phosphoinositide-interacting protein 1) (1:2000; #PA5-34973, RRID:AB_2552322), anti-GCP60 (anti-Golgi resident protein GCP60) (1:1000; #MA5-25999, RRID:AB_2723827), and anti-GRASP65 (anti-Golgi reassembly-stacking protein of 65 kDa) (1:5000; #PA3910, RRID:AB_2113207) (Thermo Fisher, Waltham, Massachusetts, USA);
- ER stress pathway: anti-p-p38 MAPK α (anti-phopsho-mitogen-activated protein kinase p38) (1:1000; #PA5-37536, RRID:AB_2554145), anti-TRAF2 (anti-TNF receptor-associated factor 2) (1:1000; #PA5-20193, RRID:AB_11152352), anti-GADD34 (anti-phosphatase 1 regulatory subunit 15A) (1:1000; #PA1139, RRID:AB_2539894), anti-p-IRE1 α (anti-phospho-unfolded protein response sensor) (1:1000; #PA1-16927, RRID:AB_2262241), anti-p-PERK (anti-phospho-translation initiation factor 2-alpha kinase 3) (1:1000; #PA5-40294, RRID:AB_2576881), anti-ATF6 (anti-activating transcription factor 6) (1:1000; #PA5-68556, RRID:AB_2688633), anti-c-ATF4 (anti-cleaved activating transcription factor 4) (1:1000; #PA5-36624, RRID:AB_2553621), anti-p-ASK1 (anti-phospho-apoptosis signal-regulating kinase 1) (1:1000; #PA5-36619, RRID:AB_2553618), anti-p-CHOP (anti-phospho-C/EBP homologous protein) (1:1000; #PA5-36796, RRID:AB_2553739), and anti-p-eIF2a (anti-phospho-translation initiation factor 2A) (1:1000; #MA5-15133; RRID: AB_10983400) (Thermo Fisher, Waltham, MA, USA).

Further, membranes were washed four times with TBST for 5 min and incubated with one of the following horseradish peroxidase-conjugated secondary antibody: anti-rabbit (1:40,000; #A0545, RRID: AB_257896) or anti-mouse (1:40,000; #A9044, RRID: AB_258431) (Sigma, Poznan, Poland). After the next four washings, the detection of blots was carried out using the ECL Western Blotting kit (BioRad, Hercules, CA, USA) and the Fusion Fx7 (Viber Lourant, Collegien, Francja) system. The densitometric analysis was performed with the GelQuantNET software (http://biochemlabsolutions.com). The bands were quantified and normalized to their corresponding β-actin bands.

### 2.4. Reverse Transcription PCR

Total RNA was isolated using Trizol reagent according to the manufacturer’s protocol (Thermo Fisher, Waltham, MA, USA). Briefly, cells were lysed in 0.5 mL of Trizol and then 200 µL of chloroform was added. After 15 min incubation in room temperature, homogenates were centrifuged (15 min, 10,000× *g*, 4 °C) and aqueous phase was transferred to a fresh tube. RNA was precipitated with 0.5 mL of isopropyl alcohol and pelleted during centrifugation (10 min, 12,000× *g*, 4 °C). The RNA pellet was washed with 1 mL of 75% ethanol and dissolved in nuclease-free H_2_O. The concentration and purity of RNA preparations were controlled on NanoDrop 2000 spectrophotometer (Thermo Fisher, Waltham, MA, USA) at optical density 260/280, while RNA integrity during standard 0.8% agarose gel electrophoresis and staining with ethidium bromide. Then, 1 µg of RNA was reverse transcribed to cDNA with High-Capacity cDNA Reverse Transcription Kit (Thermo Fisher, Waltham, MA, USA) in a total volume of 20 µL. RNA was diluted to 10 µL in nuclease-free H_2_O and mixed with 10 µL of freshly prepared master mix [2 µL 10 × RT Buffer, 0.8 µL 25 × dNTP Mix, 2 µL 10 × RT Random Primers, 1 µL Multiscribe Reverse Transcriptase (50 U) and 3.2 µL H_2_O]. The reaction was carried out as follows: 25 °C 10 min; 37 °C 120 min; 85 °C 5 min. 

Primer sequences for *XBP-1* were selected according to a prior research [10]. The PCR (polymerase chain reaction) reactions were performed in 10 µL volume containing 5 µL 2 × PCR Master Mix TaqNova-RED (DNA Gdansk, Gdansk, Poland), 2 µL of each 1 µM forward and reverse primers (Genomed, Warsaw, Poland), and 1 µL of diluted cDNA (10 ng). The amplification of PCR was carried out for 35 cycles of denaturing at 95 °C for 45 s, annealing at 57 °C for 45 s, and extending at 72 °C for 45 s, followed by a final extension at 72 °C for 10 min. PCR products were electrophoretically detected on 3% agarose gel after staining with ethidium bromide. Primers used are as follows: total variant of *XBP1*: F- GAATGAAGTGAGGCCAGTGG, R- ACTGGGTCCTTCTGGGTAGA; *ACTB*: F-CACCATTGGCAATGAGCGGTTC, R-AGGTCTTTGCGGATGTCCACGT. After visualization with the use of the Fusion Fx7 (Viber Lourant) system, the densitometric analysis was performed with the GelQuantNET software. The bands were quantified and normalized to their corresponding *ACTB* bands.

### 2.5. Enzyme-Linked Immunosorbent Assay (ELISA)

The levels of secreted interleukin 3 (IL-3), interleukin 7 (IL-7), and interleukin 2 (IL-2) were measured using ELISA kits from Thermo Scientific (#KHC0031 lot: 173401/A, #EHIL7 lot: 142062718, #EH2IL2 lot: 194427002, respectively) and strictly following manufacturer’s protocols. As LPS was shown to inhibit monocytes proliferation, the results were calculated as pg of secreted cytokines per 1000 cells [3].

### 2.6. Statistical Analysis

Data shown represent the means ± standard deviation. The experiments were carried out in at least three biological repetitions. Statistical multiple comparisons were performed using GraphPad Prism ver. 6.0 and the data were assessed with one-way ANOVA followed by Dunnett’s post hoc test. A *p*-value of < 0.05 was considered statistically significant and is displayed as: * *p* < 0.05, ** *p* < 0.01, and *** *p* < 0.001.

## 3. Results

**Figure 1:**
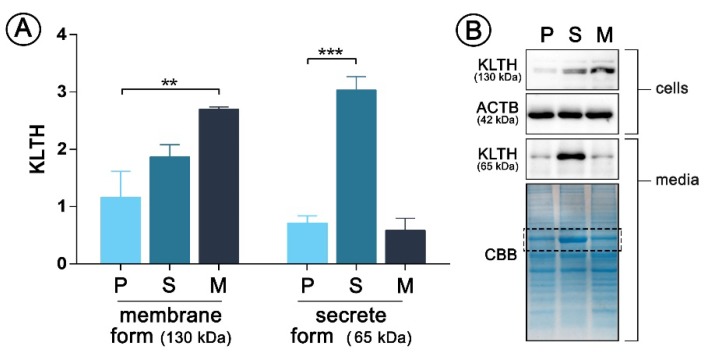
pDNA(plasmid DNA)-mediated klotho overexpression in human monocytic cells Cells were transfected with pDNA and selected in antibiotics to obtain stable clones and then Western Blot analysis of klotho membrane (130 kDa) and secrete (65 kDa) forms expression was performed (**A**). Representative images of Western Blot membrane and Coomassie staining are presented (**B**). The bands were quantified and normalized to their corresponding β-actin bands in the case of membrane form of klotho or to CBB staining in the case of secrete form. Bars indicate SD, *n* = 3, *** *p* < 0.001, ** *p* < 0.01 (one-way ANOVA and Dunnett’s a posteriori test). ACTB actin, KLTH klotho, CBB Coomassie Brilliant Blue staining.

This study is a follow-up project using the same model of monocytes stable overexpressing membrane (mKL-pDNA) and secrete (sKL-pDNA) form of klotho as described previously [3]. However, here we used a new batch of cells obtained from EACC and decided to present results of Western Blot supporting the good efficiency of transfection. As can be seen in Figure 1, the expression of membrane form of klotho with a molecular weight of 130 kDa increased by 2.32-fold (*p* < 0.01) in mKL-pDNA cells when compared to cells transfected with the control plasmid (vector cells). Simultaneously, the level of klotho secreted into cell culture media (65 kDa) by sKL-pDNA cells increased by 4.26-fold (*p* < 0.001) when compared to vector cells (Figure 1).

### 3.1. Klotho Prevents Activation of LPS-Mediated Golgi Apparatus Stress Response

**Figure 2:**
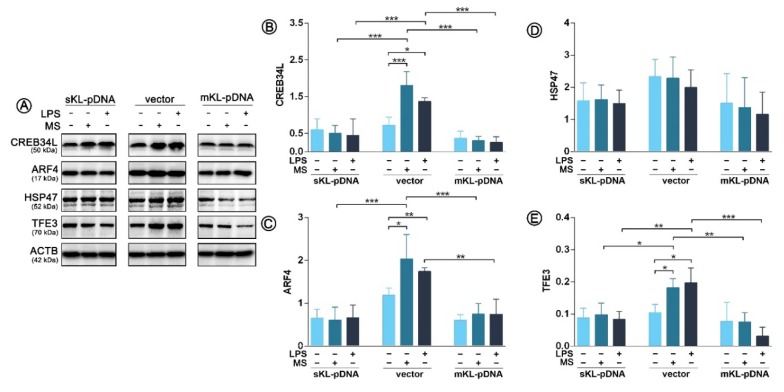
Expression of proteins involved in the Golgi stress response. Klotho expression was upregulated with pDNA, cells were treated for 72 h with monensin sodium or 168 h with LPS and expression of proteins involved in CREB34L-, HSP47- and TFE3-mediated pathways was evaluated with Western Blot method (**A**), i.e.,: CREB34L (**B**), ARF4 (**C**), HSP47 (**D**), and TFE3 (**E**). The bands were quantified and normalized to their corresponding β-actin bands. Bars indicate SD, *n* = 3, *** *p* < 0.001, ** *p* < 0.01, * *p* < 0.05, no indication – no statistical significance (one-way ANOVA and Dunnett’s a posteriori test).

**Figure 3:**
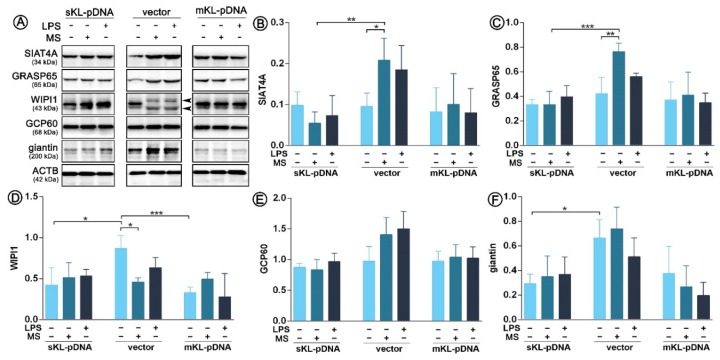
Expression of proteins involved in the Golgi-mediated stress response. Klotho expression was upregulated with pDNA, cells were treated for 72 h with monensin sodium or 168 h with LPS and the expression of TFE3-related proteins was evaluated with Western Blot method (**A**), i.e.,: SIAT4A (**B**), GRASP65 (**C**), WIPI1 (**D**), GCP60 (**E**), and giantin (**F**). The bands were quantified and normalized to their corresponding β-actin bands. Bars indicate SD, *n* = 3, *** *p* < 0.001, ** *p* < 0.01, * *p* < 0.05, no indication – no statistical significance (one-way ANOVA and Dunnett’s a posteriori test).

After establishing models of THP-1 cells overexpressing secrete or membrane form of klotho, we verified which mechanistic pathways of Golgi stress response are activated in cells due to the treatment with LPS. During the study, we observed activation of two out of three well-known pathways i.e., CREB34L and TFE3 in vector cells, which was not confirmed in cells overexpressing klotho, secrete or membrane form. As confirmed by Western Blot method, treatment with LPS in vector cells resulted in a 2.25-fold (*p* < 0.05) upregulation of CREB34L transcription factor expression (Figure 2A,B), which was further followed by a 1.77-fold (*p* < 0.01) increase in transcriptional induction of ARF4 (Figure 2A,C). Treatment with monensin sodium as a positive control resulted in similar effects. On the other hand, in klotho-overexpressing cells, the levels of both factors, i.e., CREB34L and ARF4, remained unaffected. Further, we did not confirm the activation of HSP47-mediated pathway in any of the analyzed set-ups; surprisingly also in monensin sodium-treated vector cells (*p* > 0.05) (Figure 2A,D). The activation of the TFE3 pathway was confirmed, but only in vector cells (Figure 2A,E, Figure 3). As shown on Figure 2E, LPS and monensin sodium treatment resulted in a 2.93- and 3.46-fold (*p* < 0.05) upregulation of TFE3 level in vector cells, respectively. An overexpression of the secrete form of klotho resulted in 54.1% downregulation of TFE3 expression in monocytes due to the LPS treatment (*p* < 0.01), while in cells overexpressing membrane form of klotho there was a 80.2% decrease. A similar tendency was revealed for monensin sodium-stimulated cells (*p* < 0.001) (Figure 2A,E).

As we confirmed activation of TFE3 transcription factor, we decided to analyze the synthesis pattern of Golgi-related proteins of which expression is induced by the TFE3 pathway, i.e., SIAT4A, GRASP65, WIPI1, GCP60, and giantin. As it can be seen on Figure 3, LPS treatment did not led to any alterations in the expression pattern of any of the analyzed proteins in vector or sKL-pDNA or mKL-pDNA cells. In contrast, monensin sodium stimulation upregulated the level of SIAT4Aand GRASP65 in vector cells by a 1.78- (*p* < 0.05) and 1.54-fold (*p* < 0.01), respectively. At the same moment, overexpression of the secrete form of klotho prevented these changes (Figure 3A–C). WIPI1 level was downregulated by 47.1% (*p* < 0.05) in vector cells treated with monensin sodium, perhaps due to the two band product of WIPI1 (Figure 3A,D). Simultaneously, non-treated sKL-pDNA cells were characterized by a statistically significant downregulated level of WIPI1 (*p* < 0.05; Figure 3A,D) and giantin (*p* < 0.05; Figure 3A,F) when compared to non-treated vector cells. Finally, both, LPS and monensin sodium stimulation, resulted in a slight, however not statistically significant, increase in the pool of GCP60, but only in vector cells (*p* > 0.05; Figure 3A,E).

### 3.2. Klotho Prevents Activation of LPS-Mediated Endoplasmic Reticulum Stress Response

**Figure 4:**
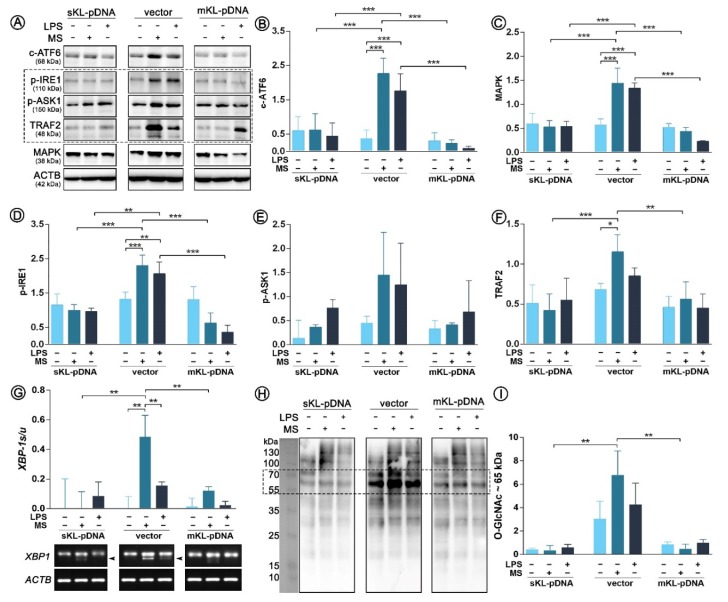
Expression of factors involved in ER stress response. Klotho expression was upregulated with pDNA, cells were treated for 72 h with monensin sodium or 168 h with LPS and expression of proteins involved in ATF6- and IRE1-mediated pathways was evaluated with Western Blot method (**A**), i.e.,: c-ATF6 (**B**), MAPK (**C**), p-IRE1 (**D**), p-ASK1 (**E**), and TRAF2 (**F**). XBP-1 splicing was evaluated by RT-PCR (**G**) and O-GlcNAc levels by WB (**H**,**I**). The bands were quantified and normalized to their corresponding β-actin bands. Bars indicate SD, *n* = 3, *** *p* < 0.001, ** *p* < 0.01, * *p* < 0.05, no indication – no statistical significance (one-way ANOVA and Dunnett’s a posteriori test).

As endoplasmic reticulum (ER) stress response is inseparably linked with Golgi stress response through ATF6 cleavage, in the next part of this study we decided to control if the course of signaling pathways of ER stress response is affected by LPS treatment. Firstly, we confirmed the increased pool of cleaved ATF6 (c-ATF6) in LPS-treated vector cells. The result was strongly statistically significant and the observed differences were 4.69- (*p* < 0.001) and 6.04-fold (*p* < 0.001) increases due to the LPS and monensin sodium stimulation, respectively. In klotho, overexpressing cells and upregulated ATF6 cleavage was not noticed (Figure 4A,B). Further, we controlled the course of the next pathway of ER stress response activated by IRE1 sensor molecule and followed by the formation of pIRE1-pASK1-TRAF2 complex. Enhanced phosphorylation of IRE1 was revealed in vector cells treated with both, LPS or monensin sodium (*p* < 0.001; Figure 3A,D). Similar observation was done for p-ASK1 levels, however due to the long error bars the differences did not become statistically significant (*p* > 0.05; Figure 3A,E). The pools of the third molecule involved in the formation of the mentioned complex were also upregulated in vector cells after treatment with LPS (1.25-fold increase; *p* > 0.05) and monensin sodium (1.69-fold increase; *p* < 0.05) (Figure 4A,F). The formation of pIRE1-pASK1-TRAF2 complex is usually followed by activation of MAPK pathway, which was also noticed in this case (Figure 4A,C). In sKL-pDNA and mKL-pDNA cells, the levels of analyzed factors remained unaffected (Figure 4A–F). On the other hand, phosphorylation of IRE1 may lead in parallel to splicing of XBP-1 to trigger unfolded protein response. The presence of sXBP-1 band on agarose gel was visualized in monensin sodium-treated (*p* < 0.01) and slightly in LPS-activated vector cells (*p* > 0.05) (Figure 4G–H). Splicing XBP-1 was probably mediated by elevated O-GlcNAcylation of proteins about 65 kDa, as shown of Figure 4H,I. Its highest levels were confirmed in vector cells treated with monensin sodium; 94% (*p* < 0.01) and 92% (*p* < 0.01) reduction when compared to monensin sodium-treated sKL-pDNA cells and mKL-pDNA cells was noticed, respectively (Figure 4H,I).

**Figure 5:**
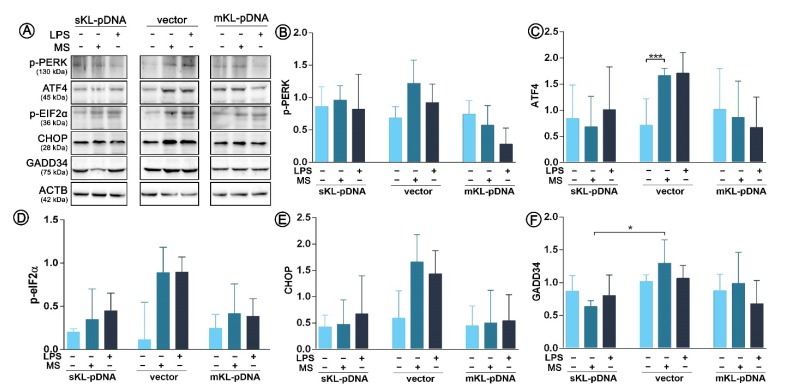
Expression of factors involved in ER stress response. Klotho expression was upregulated with pDNA, cells were treated for 72 h with monensin sodium or 168 h with LPS and expression of proteins involved in PERK-mediated pathway was evaluated with Western Blot method (**A**), i.e.,: p-PERK (**B**), ATF4 (**C**), p-eIF2α (**D**), CHOP (**E**), and GADD34 (**F**). The bands were quantified and normalized to their corresponding β-actin bands. Bars indicate SD, *n* = 3, *** *p* <0.001, * *p* <0.05, no indication – no statistical significance (one-way ANOVA and Dunnett’s a posteriori test).

Since we confirmed the activation of both ATF6 and IRE1 pathways of endoplasmic reticulum stress response, we decided to control also the third well-known branch of UPR, i.e., the PERK pathway. Surprisingly, we noticed only one statistically significant change in its course. Monensin sodium stimulation resulted in a 2.33-fold increase in ATF4 level in vector cells (*p* < 0.01; Figure 5A,C). No fluctuations in PERK phosphorylation status were noticed (Figure 5A,B). Therefore, ATF4 upregulation was probably independent of PERK pathway. Activation of eIF2α, downstream effector molecule of PERK, did not occur as assessed by evaluation of its phosphorylation profile (*p* > 0.05; Figure 5A,D). Moreover, we did not confirm activation of two target genes of eIF2α. The levels of CHOP and GADD34 remained unaffected in all analyzed set-ups (*p* > 0.05; Figure 5E,F).

### 3.3. Klotho Affects the Levels of Cytokines Directly Linked with the Golgi Apparatus Status

**Figure 6:**
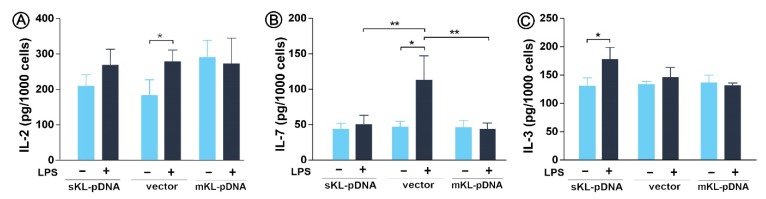
Levels of proinflammatory cytokines. Klotho expression was upregulated with pDNA, cells were treated for 72 h with monensin sodium or 168 h with LPS and secretion of IL-2 (**A**), IL-7 (**B**), and IL-3 (**C**) was evaluated with ELISA method. Bars indicate SD, *n* = 3, ** *p* < 0.01, * *p* < 0.05, no indication – no statistical significance (one-way ANOVA and Dunnett’s a posteriori test).

Finally, we decided to control whether the state of Golgi stress affects the secretion of proinflammatory cytokines, which final modification, glycosylation, and packaging take place in the Golgi apparatus. LPS treatment resulted in a 1.51-fold increase in the secretion of IL-2 in vector cells (*p* < 0.05) with slight but not statistically significant effect in sKL-pDNA (Figure 5A). A 2.36-fold (*p* < 0.05) enhancement in the level of IL-7 was also observed in vector cells stimulated with LPS. Moreover, the levels of IL-7 were downregulated by 54.5%in sKL-pDNA (*p* < 0.01) and 60.3% in mKL-pDNA cells (*p* < 0.01) after LPS activation when compared to vector cells (Figure 5B). The level of third analyzed interleukin, i.e., IL-3, was affected only in sKL-pDNA cells after stimulation with LPS and a 1.35-fold increase was reported (*p* < 0.05; Figure 6C).

## 4. Discussion and Conclusions

The present study establishes the role of klotho as a modulator of senescence-associated secretory phenotype (SASP) and reveals, for the first time, an association between klotho and Golgi, as well as ER stress response in this context. We provide evidence that the initiation of immunosenescent-like phenotype in monocytes is accompanied by activation of CREB34L and TFE3 Golgi stress response and ATF6 and IRE1 endoplasmic reticulum stress response, while klotho overexpression prevents these changes. The effect is similar for secrete and membrane form of klotho, due to the fact that the transmembrane form of klotho protein, instead of being present on the cell surface, was found to localize in the endoplasmic reticulum, endosomes, and Golgi apparatus, associating with its binding protein, α1-Na^+^/K^+^ ATPase [11].

The endoplasmic reticulum is highly active in monocytes due to the heavy engagement in the synthesis, processing, and secretion of cytokines. To date, three branches of UPR have been described: PERK, IRE1, and ATF6. The involvement of klotho in all of them has been presented in a study conducted by Banerjee et al. (2013). They reported that klotho downregulation significantly increases ER stress and activation of UPR signaling upon treatment with agents disrupting ER homeostasis by affecting ER Ca^2+^ pump and N-linked glycosylation of proteins [12]. Ameliorating effect of klotho on ER stress and its role in modulation of the unfolded protein response was described also in some other studies [13,14,15]. Moreover, the role of klotho in LPS-mediated ER stress was studied in a different cell model. In normal fibroblasts, klotho attenuation leads to activation of ER stress response mediated by eIF2α, but not an IRE1-XBP-1 pathway [16]. Here, we confirmed the activation of an IRE1-XBP-1 branch of UPR in immunosenescent monocytes, which was inhibited by klotho overexpression. The activation of an IRE1-XBP1 arm of the UPR has been shown to promote cytokine production by monocytes/macrophages activation via membrane-bound TLRs [17]. TLR4 expression is crucial for the production of IL-6, TNF-α, and IL-23 by cells experiencing this type of stress. Activation of the IRE1-XBP-1 branch via STAT3 and STAT6, which enhances the secretory phenotype of cells, as demonstrated by transcriptional reprogramming revealing upregulation of secretion-associated genes has also been reported to be induced by cytokines, such as IL-4, IL-6, and IL-10 [18,19]. Therefore, in this study we observed upregulation of proinflammatory cytokines together with the observations done in the previous paper, where LPS treatment resulted in enhanced secretion of TNF-α, IL-1β, IL-6, and IL-10 by senescent monocytes [3], which may suggest that SASP may be not only the consequence of UPR and ER stress but also its trigger on the basis of a positive feedback loop. Although the IRE1-XBP-1 pathway is considered as being pro-survival mostly through its role in ER-associated degradation process during severe ER stress conditions, IRE1 may recruit TNF receptor-associated factor 2 and apoptosis signal-regulating kinase 1, then activate c-Jun N-terminal kinase (JNK) and induce apoptosis [20,21]. IRE-1-mediated activation of inflammasome leads to increased secretion of IL-1β and further IL-1, IL-6, and IL-8 [22]. IL-1α, as a crucial proinflammatory cytokine, has been reported to play dual function during ER stress. When co-acting with CHOP, IL-1α leads to apoptotic cell death. On the other hand, when increased secretion ofIL-1α occurs with an accompaniment of mild UPR, a cellular adaptation and tissue remodeling processes are initiated [23]. Here, the levels of CHOP remained unaffected. Also, p38 MAPK (mitogen-activated protein kinase) has been shown to be involved in ER stress-induced cell death and autophagy [24] as well as to play a crucial role in stresses-induced senescence, characterized by the release of proinflammatory cytokines [25]. Mostly due to its role in the regulation of the transcriptional activity of NF-κB [26]. In our previous study, we confirmed that klotho overexpression downregulates NF-κB activity in monocytes [3] and here additionally we report its function as a downregulator of MAPK activity. Thus, this pathway may be also directly or indirectly related to the inhibition of proliferation in immunosenescent monocytes but not apoptosis, ensuring the survival of the cells [3]. This finds confirmation in our further results, where we did not confirm activation of PERK pathway at the same time. Probably this is due to the fact that IRE1 has been shown to be attenuated by PERK via RAP2 (RNA polymerase II-associated protein 2) to abort failed ER-stress adaptation and trigger apoptosis [27]. Furthermore, a recent study shows that stress-induced senescence can be promoted through the activation of an ER stress-dependent p21 signaling [28]. According to existing literature [29], our results might support a potential causative role of ER stress in terms of UPR in SASP. However, next to UPR the crucial role in production of proinflammatory cytokines may have ER overload response mediated by Ca^2+^-ROS-NF-κB pathway [3].

Most of the cytokines are secreted through classical secretory pathways, which include routing of newly synthesized proteins from the ER to the Golgi complex, sorting in the trans-Golgi network and then transportation directly to the cell surface or transit via recycling endosomes [30]. Thus, the proper condition of Golgi is crucial for the balanced secretion of cytokines. The crosstalk on the line ER-Golgi during stress response has been mostly linked with ATF6 protein. During UPR signaling, ATF6 is transported to Golgi, where it undergoes cleavage through site-1 protease (S1P) and site-2 protease (S2P). The cleavage of ATF6 releases its N-terminal domain from the membrane as a functional BiP transcription factor which then gets translocated to the nucleus of cell for activating the transcription of ATF6’ target genes [31]. Here, we observed Golgi-dependent cleavage of ATF6 in monocytes due to the LPS treatment, which was inhibited by klotho overexpression. This confirms not only involvement of klotho in the third UPR branch, but also suggests the protective role of klotho protein on alterations in Golgi functions during monocytic immunosenescence. Current literature is lacking information on this new possible characteristic of klotho. The only available reference Golgi-klotho is a paper by Wolf et al. (2014), who presented that klotho may desialylate N-glycan of TRPV5 within Golgi to promote its binding to galectins [32]. 

In this study, we provide evidence that klotho downregulates CREB34L and TFE3 stress response pathways in immunosenescent monocytes, with no effect on HSP47-mediated mechanism. The TFE3 pathway regulates the general function of the Golgi, such as structural maintenance, N-glycosylation and vesicular transport, whereas the CREB34L and HSP47 pathways regulate pro- and anti-apoptotic functions, respectively [33]. The role of klotho in HSP47-mediated cellular response was confirmed during our study, in contrast to Song et al. (2013), who presented that klotho supplementation leads to a significant downregulation of HSP47 expression [34]. Hsp47 deletion enhances secretion of some cytokines, including matrix metalloproteinase 3 (MMP3), osteoprotegerin, and pentraxin 2 [35]. Further, the TFE3 factor was shown to cooperate to promote transcriptional upregulation of numerous proinflammatory cytokines [36]. An upstream pathway connected with this signaling axis seems to be FLCN-AMPK [37]. It was reported that CREB34L is activated by ER stress and that the Golgi membrane together with S1P and S2P proteases are absorbed by the ER membrane resulting in the CREB34L cleavage [38]. In this study, the most notable difference was the reduced expression of WIPI1 in cells overexpressing secrete or membrane form of klotho. The knowledge of WIPI1 functions is limited to its interactions with phosphoinositides and its localization in both trans-Golgi and endosomal membranes, where it regulates membrane trafficking [39]. Moreover, it has been shown that WIPI1 is associated with the lysosomal degradation of cytoplasmic components during starvation-induced autophagy [40]. Since WIPI1 is strictly involved in the phosphorylation of MAPK 1/3 [41] and klotho acts as an inhibitory modulator of MAPK signaling pathways [42], it is highly likely that the role of klotho in Golgi-mediated response in immunosenescent monocytes results from this loop. However, further studies need to be conducted to elucidate fully these interactions.

In conclusion: in this study, we provide for the first time the evidence of klotho involvement in the crosstalk on the line ER-Golgi, which may, in turn, affect activation of senescence-associated secretory phenotype. This data may be useful for a novel potential target for therapy in age-related and chronic inflammatory conditions.
